# Ticagrelor is associated with periodic breathing during exercise in coronary patients undergoing cardiac rehabilitation: a retrospective observational study

**DOI:** 10.1007/s11325-026-03737-5

**Published:** 2026-07-14

**Authors:** Simon Pointel, Xavier Delavenne, Paul Bonjean, Valentine Mismetti, Jean-Michel Vergnon, Marios Froudarakis, David Hupin, Karl Isaaz, Frédéric Roche

**Affiliations:** 1 Department of Pneumology and Thoracic Oncology, CHU Saint-Etienne, Saint-Etienne, France; 2 Clinical Innovation and Pharmacology Research Unit, CHU Saint-Etienne, Saint-Etienne, France; 3 Jean Monnet Saint-Etienne University, Saint-Etienne, France; 4 SAINBIOSE DVH U1059 Inserm, J Lisfranc Faculty of Medicine, Jean Monnet University, Saint- Etienne Cedex, France; 5 VISAS Center (Ageing, Autonomic Nervous System and Sleep Disorders) – Cardiological and Respiratory Functional Exploration Department, CHU Saint-Etienne, Saint-Etienne, France; 6 Department of Clinical and Interventional Cardiology, CHU Saint-Etienne, Saint-Priest-en-Jarez, France; 7 VISAS Center – North Hospital, University Hospital of Saint-Etienne, Saint-Etienne Cedex 2, 42055 France

**Keywords:** Ticagrelor, P2Y12 inhibitor, Periodic breathing, Cardiopulmonary exercise test, Cardiac rehabilitation, Central sleep apnoea

## Abstract

**Purpose:**

Ticagrelor, a reversible P2Y12 inhibitor widely prescribed after acute coronary syndrome (ACS), induces dyspnoea and central sleep apnoea through effects on respiratory centre activity. During cardiac rehabilitation, we observed apparent ventilatory disturbances in patients receiving ticagrelor during cardiopulmonary exercise tests (CPET). This study aimed to evaluate whether ticagrelor significantly increases the risk of periodic breathing during exercise and whether this impairs functional or metabolic performance.

**Methods:**

We performed a single-centre retrospective observational study (October 2012–December 2021) including 84 coronary patients (36 ticagrelor, 48 thienopyridines) who underwent cardiac rehabilitation following angioplasty with coronary stenting. The primary endpoint was exercise-induced periodic breathing, defined as ≥ 3 consecutive oscillation cycles of VE, VO2, or VCO2 with amplitude ≥ 30% of mean values. Secondary endpoints were peak VO2, end-tidal CO2 pressure (PETCO2), and ventilatory equivalent for CO2 (EqCO2) at peak VO2. Univariable and multivariable logistic and linear regression analyses were performed (R v4.0.0).

**Results:**

Ticagrelor was independently associated with a > 7-fold increased risk of periodic breathing during exercise in both univariable (OR = 7.82, 95% CI [2.80–24.72], *p* < 0.001) and multivariable analyses (OR = 7.17, 95% CI [2.31–25.19], *p* < 0.001), after adjustment for age, BMI, LVEF, smoking, obstructive lung disease, and neurological risk. No significant effect on PETCO2 (*p* = 0.759) or EqCO2 (*p* = 0.442) at peak VO2 was observed. In multivariable analysis, patients on ticagrelor had significantly higher peak VO2 than those on thienopyridines (+ 3.17 ml/min/kg, 95% CI [1.34–4.99], *p* < 0.001).

**Conclusion:**

Ticagrelor is significantly associated with periodic breathing during exercise in coronary patients undergoing cardiac rehabilitation. However, this ventilatory disturbance does not impair gas exchange or aerobic capacity. These findings support maintaining ticagrelor therapy in patients presenting with exercise-induced periodic breathing.

## Introduction

Acute coronary syndromes (ACS) represent a major public health concern, with increasing incidence and significant morbidity and mortality [1]. Standard acute-phase management relies on coronary angioplasty with stenting and dual antiplatelet therapy combining acetylsalicylic acid with a P2Y12 receptor inhibitor [2].

Among P2Y12 inhibitors, thienopyridines (clopidogrel and prasugrel) are irreversible inhibitors requiring hepatic activation. Ticagrelor is a directly-acting reversible inhibitor that does not require metabolic conversion [3–5]. The PLATO trial demonstrated ticagrelor’s superiority over clopidogrel in reducing cardiovascular mortality, stroke, and myocardial ischaemia, substantially increasing its use in ACS management [2]. However, ticagrelor was also found to induce dyspnoea and central sleep apnoea, side effects that were previously underreported [2, 6–8].

Following ACS, patients undergo structured cardiac rehabilitation, in which physical activity plays a central role and has demonstrated significant benefits on cardiac and vascular function [9]. Physical exercise continuously stimulates the respiratory centres, which may be particularly vulnerable to pharmacological modulation by ticagrelor. Indeed, ticagrelor modulates central respiratory control through P2Y12 receptors expressed on astrocytes [10–12] and through inhibition of the ENT1 adenosine transporter, which increases cAMP-mediated activation of pulmonary C-fibres [13, 14].

In our clinical experience at the VISAS rehabilitation centre, patients on ticagrelor frequently exhibited periodic breathing patterns during CPET. The clinical significance of this observation — in particular whether it impairs exercise performance — has not been previously investigated. The primary objective of this study was to determine whether ticagrelor is associated with a higher rate of exercise-induced periodic breathing compared with thienopyridines. The secondary objective was to evaluate whether ticagrelor induces functional or metabolic limitations during CPET.

## Methods

### Study design and participants

We conducted a single-centre retrospective observational study at the VISAS Center (CHU Saint-Etienne, France) covering October 2012 to December 2021. Inclusion criteria were: coronary event requiring percutaneous coronary intervention (PCI) with stenting, and dual antiplatelet therapy comprising acetylsalicylic acid combined with either ticagrelor or a thienopyridine (clopidogrel or prasugrel). Among the 48 patients in the thienopyridine group, 15 (31.3%) received clopidogrel and 33 (68.8%) received prasugrel; no patient was treated with ticlopidine. Exclusion criteria were: surgical coronary revascularisation, and inability to estimate systolic pulmonary arterial pressure (sPAP) by transthoracic echocardiography. *Th*e study population comprised 65% STEMI and 35% NSTEMI patients. All patients underwent early coronary angiography for myocardial revascularisation (or attempted revascularisation). Troponin monitoring confirmed the diagnosis of acute coronary event in all patients at admission to the intensive cardiac care unit; however, peak troponin values were not available for all participants. There was no significant difference in the proportion of STEMI and NSTEMI patients between the ticagrelor and thienopyridine groups. The choice of antiplatelet regimen evolved over the course of the registry: patients enrolled during the early period received dual antiplatelet therapy comprising clopidogrel and acetylsalicylic acid, while those enrolled later were preferentially assigned to ticagrelor and acetylsalicylic acid. The decision regarding the specific antiplatelet regimen was primarily driven by the modalities of coronary stenting (active stent type, stent size, treatment of coronary arterial bifurcations). Beta-blocker therapy was prescribed in 86% of patients in the ticagrelor group and 84% of patients in the thienopyridine group (p = NS), and medications remained unchanged at the end of the rehabilitation programme in both groups.

### Cardiopulmonary exercise testing

All patients underwent a maximal incremental CPET on a cycle ergometer as part of their cardiac rehabilitation programme. Two experienced medical doctors specialised in exercise physiology (D.H., F.R.) used a ramp-type protocol, consisting of 2 min of warm-up at 10 W, followed by a 10-W progressive increment every minute until exhaustion. The automated metabolic system analysed respiratory gas exchange including oxygen uptake (VO2). The mean temperature and relative humidity in the testing room were 21 °C and 24%, respectively. Resting measurements were obtained after a two-minute stabilisation period to ensure steady-state values. Peak VO2 was defined as the mean VO2 over the last 30 s of maximal exercise. Maximal exercise was confirmed by a respiratory quotient (RQ) > 1.1. Lactate measurements were not routinely available in this cohort. VO2 at the first ventilatory threshold (VO2 at VT1) and peak workload (PWL) were evaluated to illustrate the achievable effort without dyspnoea and the duration of exercise, respectively. Participants were monitored continuously with electrocardiography (ECG) and heart rate (HR) was recorded throughout. Systolic blood pressure (SBP) was measured manually by an experienced nurse using a random-zero sphygmomanometer every 2 min during exercise, and at 1 and 3 min of recovery. End-tidal CO2 pressure (PETCO2) at rest and at peak VO2 was measured as a non-invasive surrogate for PaCO2.

### Endpoints

The primary endpoint was the occurrence of periodic breathing during exercise, defined as at least three consecutive oscillation cycles of the VE, VO2, or VCO2 curves with an amplitude of approximately 30% relative to their mean values, as previously described [15]. Secondary endpoints were: peak VO2 (ml/min/kg), PETCO2 at peak VO2 (mmHg), and the ventilatory equivalent for CO2 (EqCO2) at peak VO2.

### Covariates

The following potential confounders were assessed: age, body mass index (BMI), left ventricular ejection fraction (LVEF), sPAP, cardiovascular risk factors, sex, obstructive lung disease (OLD), smoking status, physical activity level, neurological risk (central neurological disorder or use of a respiratory depressant drug), and cumulative risk (glomerular filtration rate < 60 ml/min/1.73 m2, cholestasis, cytolysis, acute liver failure, or enzyme-inducing drugs that may alter ticagrelor bioavailability).

### Statistical analysis

The association between periodic breathing and ticagrelor use was assessed by Chi-square test. Associations between periodic breathing and potential confounders were evaluated using Chi-square, Student’s t-test, or Wilcoxon-Mann-Whitney tests as appropriate. Binary logistic regression was used for the primary endpoint, including variables with *p* < 0.1 in univariable analysis. Linear regression was used for continuous secondary endpoints, including variables with *p* < 0.2 in univariable analysis. All tests were two-sided with an alpha level of 0.05. No large language model (LLM) was used in data collection, statistical analysis, or manuscript preparation. Analyses were performed using R version 4.0.0.

### Ethics

This study was conducted in accordance with the Declaration of Helsinki. Ethical approval was granted by the ‘Terre d’Ethique’ Committee of CHU Saint-Etienne (Ref: IRBN892021/CHUSTE). All participants provided written informed consent. The study adheres to the STROBE reporting guidelines for observational studies (Online Resource 1).

## Results

### Study population

A total of 84 patients were analysed: 36 receiving ticagrelor and 48 receiving thienopyridines (clopidogrel: *n* = 15 [31.3%]; prasugrel: *n* = 33 [68.8%]; ticlopidine: none). The overall population was relatively young (mean age 58 years), with a mean LVEF of 57.3% and a mean sPAP of 25.75 mmHg. Both groups were comparable for most baseline characteristics (Table 1). The ticagrelor group had a significantly lower mean LVEF (54.69% vs. 59.21%, *p* = 0.034) and a significantly higher proportion of central apnoeas (25% vs. 11%, *p* = 0.04), although LVEF remained preserved in both groups. The overall population comprised 65% STEMI and 35% NSTEMI patients. The proportions of STEMI and NSTEMI did not differ significantly between the ticagrelor and thienopyridine groups. Beta-blocker therapy was used in 86% of patients in the ticagrelor group and 84% in the thienopyridine group, without significant intergroup difference (Table 1).

**Table 1:** Baseline characteristics of the study population according to P2Y12 inhibitor type

Variable	Ticagrelor (*n* = 36) Mean ± SD	Thienopyridines (*n* = 48) Mean ± SD	*p* value
Clopidogrel, n (%)	0 (0%)	15 (31.3%)	N/A
Prasugrel, n (%)	0 (0%)	33 (68.8%)	N/A
Age (years)	59.81 ± 9.4	57.44 ± 9.93	0.274
Weight (kg)	78.42 ± 13.34	79.42 ± 13.84	0.741
Height (m)	1.71 ± 0.08	1.72 ± 0.08	0.670
BMI (kg/m²)	26.82 ± 4.11	26.82 ± 3.57	0.903
LVEF (%)	54.69 ± 9.65	59.21 ± 6.61	0.034
AHI (/h)	17.89 ± 16.27	16.17 ± 13.16	0.825
% Central apnoeas	0.25 ± 0.25	0.11 ± 0.17	0.040
Central AHI (/h)	4.01 ± 6.04	2.17 ± 4.51	0.149
sPAP (mmHg)	25.39 ± 5.44	26.54 ± 7.07	0.847
Peak VO2 (ml/min/kg)	22.27 ± 5.22	20.76 ± 5.05	0.101
Peak VO2 (% theoretical)	85 ± 15	75 ± 21	0.012
VO2 at VT (ml/min/kg)	15.60 ± 5.07	14.61 ± 3.99	0.270
EqCO2 at peak VO2	37.43 ± 4.79	38.68 ± 6.83	0.501
PETCO2 at rest (mmHg)	28.68 ± 3.54	30.40 ± 4.28	0.055
PETCO2 at peak VO2 (mmHg)	30.26 ± 3.99	29.67 ± 6.13	0.835
VE at peak VO2 (% theoretical)	83 ± 15	73 ± 19	0.007
HR max (bpm)	126.14 ± 24.02	126.04 ± 22.16	0.978
RQ at peak VO2	1.11 ± 0.10	1.17 ± 0.09	0.007
GFR (ml/min/1.73 m²)	83.89 ± 17.11	86.92 ± 18.68	0.282
Beta-blocker therapy (%)	86	84	0.862

*BMI* body mass index, *LVEF* left ventricular ejection fraction, *AHI* apnoea/hypopnoea index, *sPAP* systolic pulmonary arterial pressure, *VT* ventilatory threshold, *EqCO2* ventilatory equivalent for CO2, *PETCO2* end-tidal CO2 pressure, *VE* ventilation, *HR* heart rate, *RQ* respiratory quotient, *GFR* glomerular filtration rate, *SD* standard deviation

### Primary endpoint: periodic breathing during exercise

Ticagrelor was strongly and independently associated with the occurrence of periodic breathing during CPET. In univariable analysis, the odds ratio was 7.82 (95% CI [2.80–24.72], *p* < 0.001). After adjustment for age, BMI, smoking status, LVEF, obstructive lung disease, and neurological risk, the association remained highly significant (OR = 7.17, 95% CI [2.31–25.19], *p* < 0.001) (Table 2).

**Table 2:** Univariable and multivariable logistic regression for the primary endpoint (periodic breathing during exercise)

Variable	Crude OR (95% CI)	Univariable *p*	Adjusted OR (95% CI)	Multivariable *p*
Age	1.05 (1.001–1.11)	0.044	1.02 (0.96–1.10)	0.531
BMI	1.01 (0.89–1.15)	0.914	1.03 (0.89–1.20)	0.670
LVEF	0.94 (0.88–0.998)	0.044	0.99 (0.92–1.06)	0.818
Ticagrelor vs. thienopyridines	7.82 (2.80–24.72)	< 0.001	7.17 (2.31–25.19)	< 0.001
Chronic obstructive lung disease	2.21 (0.58–8.15)	0.290	1.65 (0.33–8.40)	0.539
Smoking	0.37 (0.14–0.98)	0.043	0.40 (0.10–1.52)	0.178
Neurological risk	0.37 (0.02–2.32)	0.668	0.46 (0.02–3.70)	0.524

*OR* odds ratio, *CI* confidence interval, *BMI* body mass index, *LVEF* left ventricular ejection fraction

### Secondary endpoints

Patients on ticagrelor had significantly higher peak VO2 values compared with those on thienopyridines in multivariable analysis (+ 3.17 ml/min/kg, 95% CI [1.34–4.99], *p* < 0.001), with no significant difference in univariable analysis (*p* = 0.186) (Table 3). Ticagrelor had no significant effect on PETCO2 at peak VO2 in either univariable (+ 0.60 mmHg, 95% CI [− 1.74; +2.93], *p* = 0.592) or multivariable analysis (*p* = 0.759) (Table 4). Similarly, EqCO2 at peak VO2 was not significantly influenced by ticagrelor in univariable (*p* = 0.501) or multivariable analysis (*p* = 0.442) (Table 5).

**Table 3:** Univariable and multivariable linear regression for peak VO2 (ml/min/kg)

Variable	Crude Δ (95% CI)	Univariable *p*	Adjusted Δ (95% CI)	Multivariable *p*
Age	1.05 (1.001;1.11)—	0.044	1.02 (0.96-1.10)—	0.531
BMI	1.01 (0.89;1.15)—	0.914	1.03 (0.89-1.20)—	0.670
LVEF	0.94 (0.88;0.998)—	0.044	0.99 (0.92-1.06)—	0.818
Ticagrelor vs. thienopyridines	+ 1.51 (− 0.74; +3.75)	0.186	3.17 (1.34; 4.99)	< 0.001
Obstructive lung disease	2.21 (0.58;8.15)—	0.290	1.65 (0.33-8.40)—	0.539
Smoking	0.37 (0.14;0.98)—	0.043	0.40 (0.10-1.52)—	0.178

Δ mean difference (ml/min/kg), *CI* confidence interval, *BMI* body mass index, *LVEF* left ventricular ejection fraction. — variable included in multivariable model; crude estimate not reported separately

**Table 4:** Univariable and multivariable linear regression for PETCO2 at peak VO2 (mmHg)

Variable	Crude Δ (95% CI)	Univariable *p*	Adjusted Δ (95% CI)	Multivariable *p*
Ticagrelor vs. thienopyridines	+ 0.60 (− 1.74; +2.93)	0.592	+ 0.37 (− 2.02; +2.76)	0.759
Cardiovascular risk factors	−1.15 (− 2.35; +0.06)	0.062	−0.80 (− 2.03; +0.44)	0.205
Obstructive lung disease	+ 3.03 (− 0.33; +6.40)	0.077	+ 2.63 (− 0.84; +6.09)	0.135
Physical activity	+ 2.40 (− 0.02; +4.83)	0.051	+ 2.28 (− 0.18; +4.74)	0.069

PETCO2 end-tidal CO2 pressure, Δ mean difference (mmHg), *CI* confidence interval

**Table 5:** Univariable and multivariable linear regression for EqCO2 at peak VO2

Variable	Crude Δ (95% CI)	Univariable *p*	Adjusted Δ (95% CI)	Multivariable *p*
Ticagrelor vs. thienopyridines	−1.24 (− 3.89; +1.41)	0.501	−1.03 (− 3.69; +1.63)	0.442
Cardiovascular risk factors	+ 1.45 (+ 0.08; +2.82)	0.032	+ 1.11 (− 0.28; +2.49)	0.115
Obstructive lung disease	−3.73 (− 7.55; +0.09)	0.056	−2.49 (− 6.49; +1.52)	0.221
Smoking	−1.79 (− 4.51; +0.94)	0.171	−1.59 (− 4.31; +1.12)	0.246
Physical activity	−2.58 (− 5.34; +0.19)	0.130	−2.56 (− 5.31; +0.18)	0.067

EqCO2 ventilatory equivalent for CO2, Δ mean difference, *CI* confidence interval

## Discussion

The main finding of this study is that ticagrelor is independently associated with a > 7-fold increased risk of periodic breathing during exercise in coronary patients undergoing cardiac rehabilitation. Critically, however, this ventilatory disturbance does not impair gas exchange or maximal aerobic capacity, suggesting that it should not prompt a switch to an alternative antiplatelet agent.

The mechanisms underlying ticagrelor-induced ventilatory disturbances are well supported in the literature. P2Y12 receptors are expressed on astrocytes — glial cells that regulate respiratory centre activity [10–12]. By blocking P2Y12, ticagrelor diverts ADP towards P2Y1 receptors, elevating intracellular calcium and enhancing respiratory centre excitability, thereby promoting hyperventilation and ventilatory instability [16]. Crucially, this mechanism is specific to reversible P2Y12 inhibitors: ticagrelor must be continuously present to maintain receptor saturation, and newly synthesised astrocytic receptors are therefore continuously inhibited — a situation that does not arise with irreversible thienopyridines [1]. This pharmacological specificity explains why exercise-induced periodic breathing appears exclusive to ticagrelor in our cohort.

A second mechanism involves ticagrelor’s inhibition of the ENT1 adenosine transporter, resulting in increased cAMP generation and activation of pulmonary C-fibres [13, 14]. During exercise, the concomitant rise in body temperature further stimulates these mechanoreceptors, potentiating ticagrelor’s pro-ventilatory effects. These mechanisms are directly relevant to the clinical context of cardiac rehabilitation and are consistent with the known association between ticagrelor and central sleep apnoea [6, 7, 17].

The unexpected finding of higher peak VO2 in ticagrelor-treated patients in multivariable analysis most likely reflects residual confounding rather than a pharmacological benefit on aerobic capacity. The ticagrelor group had a higher peak VO2 as a percentage of theoretical values (85% vs. 75%, *p* = 0.012) and higher ventilatory equivalents at peak exercise, consistent with a better-preserved cardiorespiratory reserve. This interpretation is supported by the absence of any effect on gas exchange markers. The maximal nature of exercise was confirmed in both groups by a respiratory quotient (RQ) target > 1.1, with no significant difference in RQ max between groups, supporting the validity of peak VO2 comparisons. The key clinical conclusion is therefore that ticagrelor does not metabolically limit patients during CPET [18].

From a clinical standpoint, these findings are immediately relevant to cardiac rehabilitation practice. When periodic breathing is observed during CPET in a patient receiving ticagrelor, the reflex consideration of switching to a thienopyridine may not be justified. Ticagrelor’s superiority over clopidogrel for reducing cardiovascular mortality and ischaemic events, demonstrated in the PLATO trial [2], constitutes a robust argument for maintaining therapy. Furthermore, switching antiplatelet agents exposes patients to the risk of stent thrombosis, which has been specifically reported in the context of ticagrelor discontinuation for dyspnoea [19]. The benefit-risk balance clearly favours maintaining ticagrelor in patients presenting with exercise-induced periodic breathing without metabolic impairment.

The finding of a significantly higher proportion of central apnoeas in the ticagrelor group (25% vs. 11%, *p* = 0.04) reinforces the concept that ticagrelor destabilises respiratory control not only during exercise but also during sleep, consistent with multiple prior reports [6, 7, 17]. The mechanisms underlying this dual nocturnal and exertional ventilatory destabilisation remain incompletely understood [20–24]. In our cohort, the subgroup of patients presenting with both central sleep apnoea and exercise-induced periodic breathing did not differ significantly in peak aerobic capacity from patients with exercise-induced periodic breathing alone, suggesting that the concurrent presence of central sleep apnoea does not further impair functional capacity in this context. However, not all patients underwent polysomnography or ventilatory polygraph, which limits the strength of this conclusion. The prognostic impact of ticagrelor-related central sleep apnoea, which is often transient (limited to the period of dual antiplatelet therapy), also remains uncertain [24]. Nevertheless, the demonstrated reduction in stent restenosis and thrombosis risk associated with ticagrelor-based dual antiplatelet therapy remains a critical consideration [8], and the benefit-risk balance continues to favour maintaining ticagrelor even in the presence of both nocturnal and exertional ventilatory control instability.

Several limitations merit discussion. The retrospective design limits causal inference, and the absence of randomisation and blinding introduces selection bias. Periodic breathing detection during CPET ideally requires a minimum resting baseline of five minutes, which was not systematically applied; this may have led to underdetection of the primary endpoint. For statistical power reasons, clopidogrel and prasugrel were pooled as a single thienopyridine comparator — a three-arm analysis would have been more informative. Finally, as a single-centre study with a limited sample size, the external validity of our findings requires confirmation in larger, prospective, multicentre studies.

## Data Availability

The datasets analysed during the current study are not publicly available owing to patient confidentiality requirements but are available from the corresponding author on reasonable request.
